# IFCC methods for the measurement of catalytic concentration of enzymes. Part 8. IFCC method for lactate dehydrogenase (L-lactate: NAD^+^ oxidoreductase, EC 1.1.1.27)

**DOI:** 10.1155/S1463924694000210

**Published:** 1994

**Authors:** Renze Bais, Margaret Philcox

**Affiliations:** Division of Chnical Biochemistry Institute of Medical and Veterinary Science frome Road Adelaide South Australia 5000 Australia

## Abstract

Human lactate dehydrogenase is a tetramer made up of two types of subunits, either H (heart) or M (muscle). Combination of these subunits gives rise to the five isoenzymes of lactate dehydrogenase which are found in mammalian tissues. The relative proportions of the individual isoenzymes found in serum of patients is related to the severity of the lesion in the organ or tissue from which they originate and the half-life of the individual tissue-specific enzymes. Thus, one cannot predict the relative proportions of the different isoenzymes in any one patient sample.

Lactate dehydrogenase catalyses the reversible oxidation of lactate to pyruvate and either reaction can be measured readily. However, in this method, the lactate to pyruvate reaction has been selected because of the following reasons; the time-course of the reaction is more linear, the reaction results in an increase in absorbance and optimization of substrates is possible (see appendix A).

The principles applied in the selection of the conditions of measurement are those stated in previous publications by the IFCC’s Committee on Enzymes [1]. Human serum and tissue extracts have been used as the sources of enzymes. The final concentration of substrates and the pH have been selected on the basis of experiments and empirical optimization techniques and have been confirmed by calculation from rate equations. The catalytic and physical properties of the isoenzymes differ, but because of the importance of the heart specific isoenzyme (LD1) in the assessment of coronary heart disease and as a tumour marker, this method has been optimized for this isoenzyme. However, the method is also suitable, although less optimally, for the determination of the other isoenzymes of lactate dehydrogenase which may be present in serum.


Journal of Automatic Chemistry, Vol. 16, No. 5 (September–October 1994), pp. 167–182

### IFCC methods for the measurement of catalytic concentration of enzymes

### Part 8. IFCC method for lactate dehydrogenase (L-lactate: $NAD^+$ oxidoreductase, EC 1.1.1.27)

**Renze Bais*** **and Margaret Philcox**

*Division of Clinical Biochemistry, Institute of Medical and Veterinary Science, Frome Road, Adelaide, South Australia 5000, Australia*

*Human lactate dehydrogenase is a tetramer made up of two types of subunits, either H (heart) or M (muscle). Combination of these subunits gives rise to the five isoenzymes of lactate dehydrogenase which are found in mammalian tissues. The relative proportions of the individual isoenzymes found in serum of patients is related to the severity of the lesion in the organ or tissue from which they originate and the half-life of the individual tissue-specific enzymes. Thus, one cannot predict the relative proportions of the different isoenzymes in any one patient sample.*

*Lactate dehydrogenase catalyses the reversible oxidation of lactate to pyruvate and either reaction can be measured readily. However, in this method, the lactate to pyruvate reaction has been selected because of the following reasons; the time-course of the reaction is more linear, the reaction results in an increase in absorbance and optimization of substrates is possible (see appendix A).*

*The principles applied in the selection of the conditions of measurement are those stated in previous publications by the IFCC's Committee on Enzymes [1]. Human serum and tissue extracts have been used as the sources of enzymes. The final concentration of substrates and the pH have been selected on the basis of experiments and empirical optimization techniques and have been confirmed by calculation from rate equations. The catalytic and physical properties of the isoenzymes differ, but because of the importance of the heart specific isoenzyme (LD1) in the assessment of coronary heart disease and as a tumour marker, this method has been optimized for this isoenzyme. However, the method is also suitable, although less optimally, for the determination of the other isoenzymes of lactate dehydrogenase which may be present in serum.*

### Principle

The proposed method for the measurement of the catalytic concentration of lactate dehydrogenase in serum is based on the principles outlined by Wacker *et al.* [2] and Vanderlinde [3]. Modifications include optimization of the substrate concentrations and pH and the choice of N-methyl-D-glucamine as the buffer.

The reversible reaction catalysed by lactate dehydrogenase is as follows:

$$L(+)\text{-lactate} + NAD^+ \Leftrightarrow \text{pyruvate} + H^+ + NADH \quad (1)$$

The reaction is monitored by following the reduction of $NAD^+$ at 339 nm. The equilibrium favours the formation of pyruvate at pH values greater than 9. The enzyme exhibits absolute specificity for the L(+) isomer. L(+)-α-Hydroxybutyrate is also a substrate as are some of the higher α-hydroxy acids [4].

### Optimal conditions for measurement

These IFCC conditions are optimized reaction conditions which are defined [1] as those conditions that are most favourable for both the kinetic reactions and the technical aspects of the measurement, i.e. these conditions do not necessarily provide maximum activity.

The reaction is initiated by the addition of $NAD^+$

| | |
|---|---|
| Temperature | 30·00 ± 0·05°C |
| pH (30°C) | 9·40 ± 0·05 |
| N-Methyl-D-glucamine | 325 mmol/l |
| L(+)-Lactate | 50 mmol/l |
| Volume fraction of sample | 0·05 (1:21) |
| $NAD^+$ | 10 mmol/l |

### Instrumentation and equipment

A recording spectrophotometer suitable for accurate measurements at the wavelength of 339 nm with a constant temperature cuvette compartment must be used. The specifications for the equipment (spectral band width, light path, accuracy of temperature) should meet those of previous recommendations [1].

### Reagents

(1) N-methyl-D-glucamine, $C_7H_{17}NO_5$, *Mr* 195.22.

(2) L(+)-Lactic acid; lithium salt, $C_3H_5O_3Li$, *Mr* 96·01.

(3) β-Nicotinamide-adenine dinucleotide, free acid ($NAD^+$), $C_{21}H_{27}N_7O_{14}P_2$, *Mr* 663·4.

* *To whom correspondence should be addressed.*

© 1994 International Federation of Clinical Chemistry (IFCC)

(4) Hydrochloric acid, HCl, *Mr* 36.46 (1 mol/l and 5 mol/l).

(5) Sodium chloride, NaCl, *Mr* 58·44 (150 mmol/l).

### Purity of reagents

The assessment of reagent purity is made on the basis of functional (performance of reagent), chemical (analytical evaluation) and instrumental (absorbance or fluorescence) characteristics.

To prevent the growth of microorganisms in solutions, sterilized containers should be used. All solutions should be prepared in calibrated flasks with water meeting the following standards [5]: electrical resistivity: $\geqslant 2{\cdot}0 \times 10^4$ ohm m at 25°C; pH: 6·0–7·0; and silicates <0·1 mg/l.

### Preparation of solutions

(I) Buffer. N-methyl-D-glucamine buffer (379·2 mmol/l, pH 9·4 at 30°C)

Dissolve 74·03 g N-methyl-D-glucamine in approximately 800 mL water, adjust pH to 9·4 at 30°C with HCl. Adjust the volume to 1000 mL with water.

(II) Buffered Substrate solution. N-methyl-D-glucamine/lithium lactate (N-methyl-D-glucamine buffer 379·2 mmol/l, pH 9·4 containing 58·3 mmol/l lithium lactate).

Transfer 80 mL of N-methyl-D-glucamine buffer solution I to a 100 ml beaker containing a magnetic stirrer. Dissolve with stirring at room temperature, lactic acid, lithium salt, 0·560 g. Adjust the pH, if necessary, to 9.4 at 30°C with HCl, 1 mol/l.

Transfer the solution to a 100 ml volumetric flask and add N-methyl-D-glucamine buffer solution I to a final volume of exactly 100 ml.

(III) Reagent solution $NAD^+$/water ($NAD^+$, 105 mmol/l in water).

Dissolve 0·697 g of $NAD^+$ in 8 ml of distilled water in a 10 ml volumetric flask. When dissolved add water to a final volume of exactly 10 ml.

(IV) Solution of sodium chloride (154 mmol/l).

Dissolve 0·9 g of sodium chloride in 100 ml of water.

### Stability of solutions

Solutions I and II should be stored either in a refrigerator at 4°C or in a freezer at −20°C. Solution III can be stored at 4°C or −20°C (see Appendix A). No measureable decrease in catalytic activity of lactate dehydrogenase in serum pools is observed when reagent II and III are stored at 4°C for at least 12 days. At room temperature (20–25°C) bacterial contamination of solutions I and II is a limiting factor.

### Specimen procurement and stability

Serum is the preferred specimen. Plasma may be contaminated with platelets which contain high concentrations of lactate dehydrogenase and should be avoided. Collect blood by venipuncture with minimal manipulation and stasis. Avoid haemolysis to minimize interference by erthrocyte lactate dehydrogenase. Cell-free serum should be obtained by centrifuging clotted blood for 10 min at a relative centrifugal force of approximately 1000 g.

The stability of the lactate dehydrogenase isoenzymes varies [6].

### Measurement

### *Measurement conditions*

Wavelength: 339 nm (±1 nm).
Bandwidth: ⩽2 nm.
Light path: 10·0 ± 0·01 mm.
Final volume of reaction mixture: 3·15 ml.
Temperature: 30·0 ± 0·05°C (thermostated cuvette compartment).

### *Handling of solutions*

Before solutions can be pipetted, the temperature of reagent solutions and of specimen must be brought to the calibration temperature of the pipettes. However, use of other temperatures results in a relative error of only 0·000025 for each °C difference from the pipette calibration temperature and for most situations this error is negligible.

During the preincubation period the solution in the cuvettes must attain a temperature of 30·0 ± 0·05°C before initiating the reaction.

### *Procedures that constitute one measurement*

### *Kind of reactions*

*Table 1. Composition of reaction mixtures A, B, and C needed for one measurement of rate of conversion.*

| Kind of reaction | Sample | Solution |
|---|---|---|
| (*a*) Overall reaction | Serum | II and III |
| (*b*) Reagent blank reaction | Reagent grade water | II and III |
| (*c*) Sample blank reaction | Serum | I and III |

*Overall reaction*

*Table 2. Analytical system for measurement of the overall rate of conversion.*

| Pipette into the cuvette | Volume | Substance concentration in final complete reaction mixture | |
|---|---|---|---|
| Solution II | 2·70 ml | N-methyl-D-glucamine<br>Lithium lactate | 325 mmol/l<br>50 mmol/l |
| Serum | 0·15 ml | Volume fraction | 1:21 |
| Mix carefully, avoiding the loss of any volume of the mixture. Incubate the reaction mixture at 30°C and wait for a minimum of 180 s for temperature equilibration. Before the following step, solution III should be at 30°C. | | | |
| Solution III | 0.30 ml | $NAD^+$ | 10 mmol/l |
| Mix again and wait 30 s. Monitor the increase in absorbance at 339 nm as a function of time, for at least an addition time of 240 s. | | | |

*Reagent blank*

The same procedure as described above in table 2 is followed for the measurement of the reagent blank reaction except that reagent grade water is substitued for serum. The reagent blank is approximately 0·05 μkat/l (3 U/l).

*Sample blank*

A sample blank is determined for each sample by substituting solution I for solution II and following the same procedure as described above in table 2. Experimental evidence has shown that the sample blank can be up to 10% of the total catalytic activity. However, this appears to be due to lactate present in the sample (see Appendix A) and thus does not enter directly into the calculation of the results (see below: 'Corrections for blank reactions').

*Measurement interval*

The values of A/s of the overall lactate dehydrogenase reaction (A) are constant over a period of at least 240 s for sera with catalytic concentration of lactate dehydrogenase up to 10 ukat/l (600 U/l). Occasionally, the value of A/s may not be constant over the time period, however, if the conditions as described are followed, this is insignificant. If the absorbance change is greater than 0·0030/s, the serum sample must be diluted with solution IV and the measurement repeated. The period of observation of the blank reactions should be the same as for the overall reaction.

*Corrections for blank reactions*

The rate of the overall reaction (A) is corrected for the reagent blank value (B) as follows:

$$a_{corrected} = a_A - a_B$$

The subscripts A and B refer to the composition of the reaction mixtures referred to in table 1. The corrected value of $a$ is used in the following calculations. It equals the true rate of conversion catalyzed by lactate dehydrogenase.

The same blank does not enter into the calculations as it is considered to be due to the presence of lactate in the sample (see Appendix A). The amount of lactate contributed by the serum sample to the overall lactate concentration in the reaction mixture does not affect the rate of conversion by lactate dehydrogenase.

*Calculation*

The molar absorption coefficient, ε of NADH (30°C, 339 nm) is 630 m²/mol [7, 8]. The light path length, l, is 0·01 m (= 10 mm). Let the increase in absorbance per second at 339 nm be $a$/s. The total volume, V, is $3{\cdot}15 \times 10^{-3}$ l. The sample volume, v, is $0{\cdot}15 \times 10^{-3}$ l.

$$b = a \cdot \frac{3{\cdot}15 \times 10^{-3}}{630 \,.\, 0{\cdot}01 \,.\, 0{\cdot}15 \times 10^{-3}} \cdot \frac{s^{-1} l}{m^2 \,.\, mol^{-1} \,.\, m \,.\, l}$$

$$= a \cdot \frac{3{\cdot}15}{0{\cdot}945} \cdot \frac{Mol}{M^{-3}} \cdot s^{-1}$$

$$= a \,.\, 3{\cdot}333 \text{ mol} \,.\, s^{-1} \,.\, m^{-3}$$

$$= a \,.\, 3{\cdot}333 \text{ kat} \,.\, m^{-3}$$

$$= a \,.\, 3333 \text{ μkat/l}$$

Note, this value was calculated for a measuring time of 1 s.

Let the increase in absorbance per 60 s at 339 nm be A. $(60\text{ s})^{-1}$

$$b = A \,.\, 3333 \text{ umol} \,.\, (60\text{ s})^{-1} \,.\, l^{-1}$$

$$= A \,.\, 3333 \text{ U/l}$$

### Analytical variability

The intra-batch imprecision of the method has been determined by repeated measurement at three LD levels (table 3).

*Table 3. Intra-batch imprecision.*

| Sample | Number | Mean | S.D. | CV (%) |
|---|---|---|---|---|
| Low | 20 | 137·9 | 1·33 | 0·96 |
| Medium | 18 | 310·0 | 2·98 | 0·97 |
| High | 20 | 469·9 | 4·58 | 0·97 |

An international transferability study is being undertaken.

### Reference ranges

Reference ranges at 30°C have not been determined for this method. However, a reference range has been determined at 37°C on healthy people selected from recruiting/entrance examinations at the Medical University of Lübeck, Germany. The results are shown in table 5 of the Appendix.

### References

1. Bowers, G. N., Jr, Bergmeyer, H. U., Horder, M. and Moss, D. W., *Clinica Chimica Acta*, **98** (1979), 163.
2. Wacker, W. E. C., Ulmen, D. D. and Vallee, B. L., *New England Journal of Medicine*, **255** (1956), 449.
3. Vanderlinde, R., *Annals Clinical Laboratory Science*, **15** (1985) 13.
4. McComb, R. B., edited by Homburger, H. A. (College of American Pathologists, Skokie, 1983).
5. NCCLS Document C3-A2, Approved Guideline (1991) Villanova, PA. National Committee for Clinical Laboratory Standards 11(13).
6. Maekawa, M., *Journal of Chromatography*, **429** (1988), 373.
7. Ziegenhorn, J., Senn, M. and Bucher, T., *Clinical Chemistry*, **22** (1976), 151.
8. Bowers, R. B., Jr., *Clinical Chemistry*, **22** (1976), 177.

### Appendix A: Description of pertinent factors in obtaining optimal conditions for measurements

### Introduction

After considerable discussion, the IFCC Committee on Enzymes decided that because of the clinical importance of the heart specific isoenzyme, LD1, the reference method for lactate dehydrogenase would be optimized for this isoenzyme. However, we are mindful that this method will also be used to measure the other isoenzymes of lactate dehydrogenase, and, although the method is not necessarily optimized for these, it is still suitable for use in their measurement. The other decision that was made was that the preferred reaction direction to be assayed was from lactate to pyruvate. Many workers have shown that the lactate dehydrogenase reaction curve is curvi-linear; however, the lactate to pyruvate reaction is less curved [1, 2] than the pyruvate to lactate reaction and should be suitable for meaningful kinetic experiments. In addition, the lactate to pyruvate reaction results in an increase in absorbance and is less susceptible to substrate inhibition [3]. A number of groups have also presented preliminary results to the Committee on Enzymes suggesting that there were problems in optimizing the pyruvate to lactate reaction including unusual kinetics (two Km values for pyruvate) and difficulties in interpreting the Surface Response Optimization data.

This Appendix is a review of the various alternatives considered in choosing the final conditions. These experiments have been carried out using either serum, LD1 prepared from serum using the Roche Isomune LD1 kit, serum from patients with heart disease (LD1 serum) or liver disease (LD5 serum) or LD1 and LD5 prepared from human tissue using the method of Clark *et al.* [4]. All experiments described have been carried out at 30°C unless otherwise stated.

The experiments described in this Appendix have been carried out at buffer concentrations of 150, 250 and 325 mmol/l. A buffer concentration of 150 mmol/l was used in some early experiments to determine the optimal pH and kinetic constants. Other experiments were performed using a buffer concentration of 250 mmol/l. Results available later from the Response Surface Optimization analyses indicated an increase in buffer concentration to 325 mmol/l was necessary to achieve optimal assay conditions. Although there was a slight increase in buffering capacity at 325 mmol/l, little change in lactate dehydrogenase activity was found. However, many experiments previously performed using a 250 mmol/l buffer concentration were repeated at the optimal concentration of 325 mmol/l in order to verify that there was no change in the optimal concentrations of each reaction component.

### Buffer selection

Previous work by Buhl *et al.* [5], the Deutsche Gesellschaft für Klinische Chemie (DGKC) in Germany and by members of the Committee on Enzymes examined a variety of buffers that could be considered as suitable for the LD1 assay. These experiments included pH profile studies and examining the linearity of the reaction progress curve. From these studies, we concluded that the buffers that warranted further consideration were diethanolamine (DEA), 2-amino-2-methyl-1-propanol (AMP) and N-methyl-D-glucamine (NMG). DEA was not chosen because it can act as a substrate for alcohol dehydrogenase (up to 38% of the activity compared with using ethanol as the substrate) which can be present in some sera at high concentrations [6]. This activity can be substantial and give falsely elevated results. In some publications, this activity has been erroneously referred to as 'LD6 activity' [7–10]. Although this is also a problem with AMP (up to 20% of the alcohol dehydrogenase activity measured with ethanol as the substrate), this buffer has been compared with NMG because it has been recommended by the DGKC as the buffer of choice [11].

*Blank reactions*

In experiments by the DGKC, NMG was rejected because it gave a blank reaction whereas AMP did not. We have repeated these experiments and initially measured the blank reaction of the reagent in the absence of sample (figure 1).

These reaction rates represent a blank of 0·02 μkat/k (1·4 U/l) for AMP and 0·04 μkat (2·6 U/l) for NMG. In both cases the blank would be considered insignificant when routinely measuring the total lactate dehydrogenase

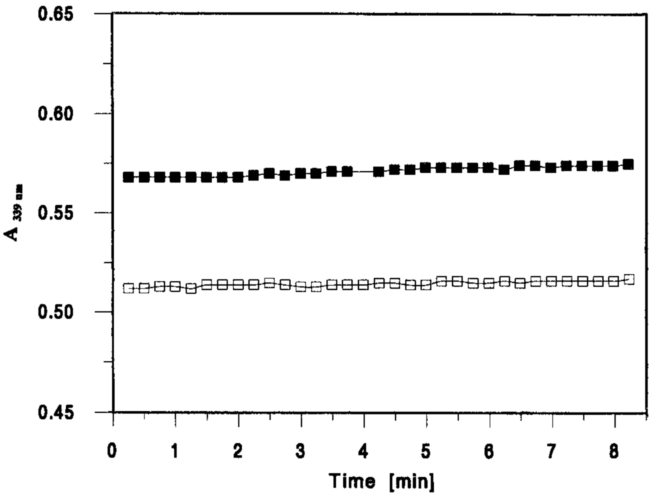

*Figure 1. Blank reactions with NMG buffer (■) and AMP buffer (□). The reaction conditions were such that when the various components were combined the final concentrations were 325 mmol/l buffer, pH 9·4, 50 mmol/l lithium lactate and 10 mmol/l NAD$^+$. Water was substituted for sample and the reaction was measured at 30°C.*

activity although they should be measured when assigning values to reference material.

*Sample blank*

The sample blank was measured with NAD$^+$ omitted from the reaction mixture (figure 2).

As can be seen in figure 2, there was no significant reaction with either NMG or AMP buffer.

The rate with NAD$^+$ included in the reaction but lactate omitted was then measured (figure 3). Although lactate was omitted, there was a significant blank reaction with both buffers giving similar results. It is postulated that

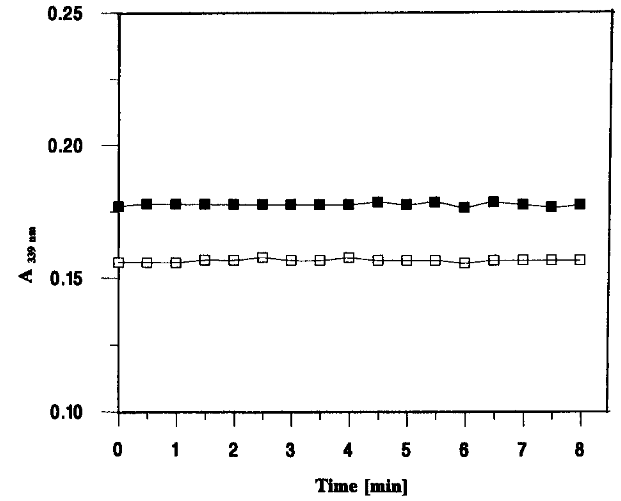

*Figure 2. Sample blank for serum containing predominantly LD1 with NMG buffer (■) and AMP buffer (□) with NAD$^+$ omitted from the reaction mixture. The reaction conditions were 250 mmol/l buffer, pH 9·4, 50 mmol/l lactate and NAD$^+$ omitted. The reaction was initiaited by adding sample.*

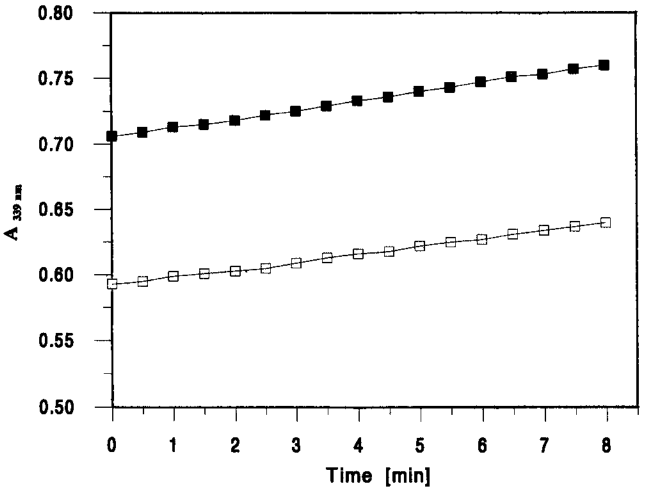

*Figure 3. Sample blank for serum containing predominantly LD1 with NMG buffer (■) and AMP buffer (□) with lactate omitted from the reaction mixture. The reaction mixture was the same as described in figure 1 except that lactate was omitted. The reaction was initiated by adding NAD$^+$.*

this reaction is due to the presence of lactate in the sample (normal range for lactate in serum is 0·2–2·0 mmol/l).

This was confirmed by adding oxamate (which is a specific inhibitor of lactate dehydrogenase) to the reaction mixture [12]. At an oxamate concentration of 100 mmol/l, the reaction as described in figure 3 (that is in the absence of added lactate) was effectively eliminated. This indicates that lactate dehydrogenase is responsible for this blank reaction and must be acting on endogenous lactate present in the sample. It does not preclude, however, lactate dehydrogenase acting on another substrate present in the serum, but we feel this is unlikely given the specificity of this enzyme.

Because the amount of lactate contributed by the sample is insignificant compared with the concentration of lactate already present in the assay mixture, its effect on measuring total lactate dehydrogenase activity will be negligible.

*Effect of temperature on buffer pH*

Although the reference method is being optimised at 30°C, many laboratories will be carrying out their assays at 37°C and thus we have studied the effect of temperature on the pH of the buffer (figure 4). This is also important because buffers are often prepared at a particular temperature and then used at another. If the effect of the temperature on the pH was not taken into account, the final assay pH would be incorrect.

It was calculated from the results that NMG changes 0·016 pH units per °C and AMP 0·021 pH units per °C indicating that AMP is affected slightly more by changes in temperature than is NMG.

The results of these experiments show there is little difference berween NMG and AMP. However, NMG is preferred as it is a non-toxic, white crystalline powder

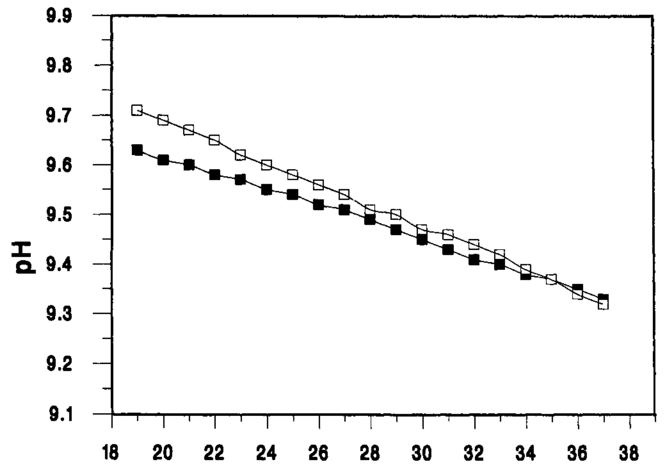

*Figure 4. Effect of temperature on the pH of NMG (■) and AMP (□) buffer. The concentration of each buffer was 325 mmol/l.*

and is thus more easily handled than AMP. Also, NMG does not act as a substrate for alcohol dehydrogenase as is the case for AMP. The properties of NMG as a buffer have been discussed previously in relation to alkaline phosphatase [13].

*Optimum pH*

To determine the optimum pH of lactate dehydrogenase, the activity was measured between pH 8·0 and 11·0. Preliminary experiments showed that the optimum pH for LD1 was greater than pH 10. However, because the method would also be used for the measurement of other isoenzmes besides LD1 and because of the increased instability of $NAD^+$ at higher pH values, this pH was

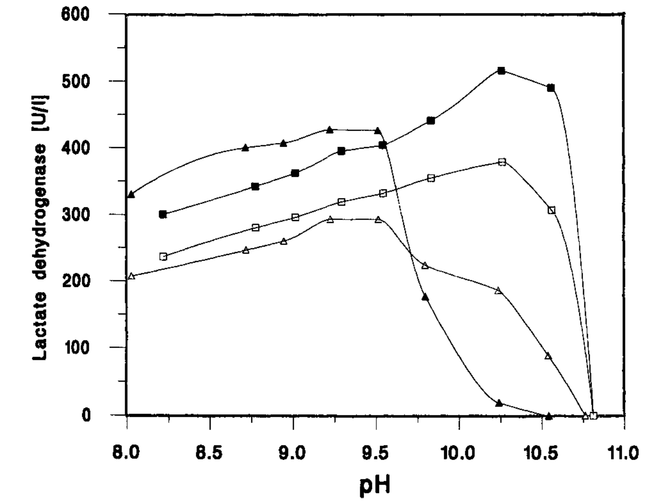

*Figure 5. Effect of varying pH on the activity of lactate dehydrogenase at 30°C. The reaction was carried out at the following concentrations, buffer, 150 mmol/l; $NAD^+$, 10 mmol/l and lactate, 50 mmol. The reaction was initiated by the addition of $NAD^+$. The samples that were assayed were purified LD1 (■), purified LD5 (▲), a specimum containing predominantly LD1 (□) and a specimen containing predominantly LD5 (△).*

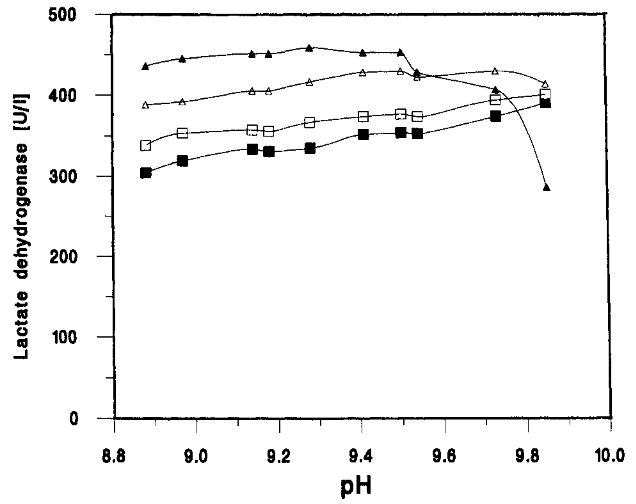

*Figure 6. Effect of varying the pH between 8·9 and 9·9 on the activity of lactate dehydrogenase activity measured at 30°C. The concentration of the reaction components were as described in figure 5 and the reaction was initiated by the addition of $NAD^+$. The samples assayed were purified LD1 (■), purified LD5 (▲), a specimen containing predominantly (LD1) (□) and a specimen containing predominantly LD5 (△).*

considered to be too high for general use. In addition, we demonstrated that LD5 was unstable at pH values greater than 9·6.

The pH profile has been examined using purified LD1 and LD5 and serum containing predominanelty LD1 (LD1 serum) and another containing predominantly LD5 (LD5 serum).

As can be seen from these results, the pH optimum is different for the various samples that were assayed (figure 5). Both purified LD5 and the serum containing LD5 reach a definite optimum between pH 9·2 and 9·5. Purified LD1 and the serum containing LD1 almost reach an optimum in the same region but then at pH values greater than 9·6, the activity increases again reaching a true optimum at 10·3. However, pH 10·3 is incompatible with the stability of $NAD^+$ and, therefore, not suitable for consideration as the assay pH. At pH values greater than 10·5, the activity of LD1, and the two serum samples decrease rapidly probably due to the instability of $NAD^+$ and the enzyme itself at these high pH values. The purified LD5 is unstable at considerably lower pH values. The region between 9·2 and 9·5, in which the activity for all the isoenzymes only increases gradually, was investigated in greater detail.

To investigate this pH region, a similar experiment as described above was carried out except that the pH range being investigated was restricted to between 8·9 and 9·9 (figure 6). The reaction was initiated by the addition of $NAD^+$ and the pH profiles of purified LD1 and LD5 and serum containing either predominantly LD1 or LD5 investigated.

The results show there is little change in the activity of the four samples when assayed between pH 9·15 and 9·55. The choice of 9·40 ± 0·05 at 30·0 ± 0·05°C as the assay pH lies within this range. Above pH 9·60, the activity of LD1 increases significantly, whereas there is a decrease

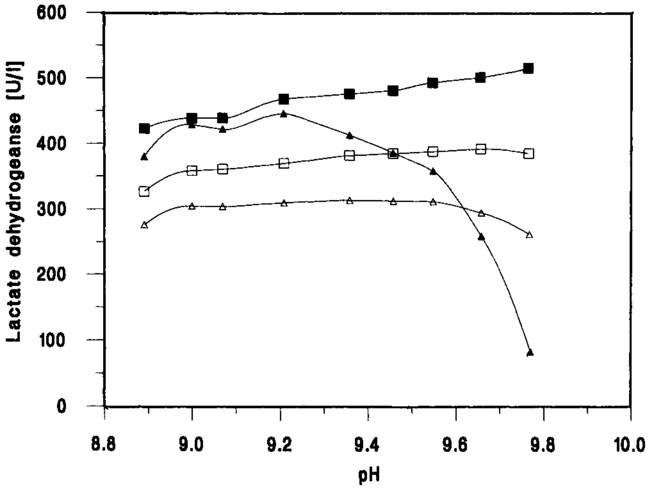

*Figure 7. Effect of varying the pH between 8·9 and 9·8 on the activity of lactate dehydrogenase activity measured at 37°C. The concentration of the reaction components were as described in figure 5 and the reaction was initiated by the addition of NAD$^+$. The samples assayed were purified LD1 (■), purified LD5 (▲), a specimen containing predominantly LD1 (□) and a specimen containing containing predominantly LD5 (△).*

in the activity of purified LD5. The serum form of LD5 is much more stable and maintains its activity up to a pH value of 9·75.

Although the IFCC chose 30°C as the assay temperature for its methods, many national societies have recommended 37°C as the preferred assay temperature. This is now the most widely used temperature for the routine assay of enzymes. The effect of pH on the activity of lactate dehydrogenase at 37°C was also investigated but only over the narrow pH range of 8·9 to 9·8 (figure 7).

The results at 37°C show that there is little change in activity for purified LD1, serum LD1 and serum LD5 when assayed between 9·20 and 9·45. Again, for LD1 there is a significant increase in activity above pH 9·55. The loss of activity of purified LD5 occurs at a lower pH but this may be due to the increased sensitivity of the enzyme to the higher assay temperature used. No such loss in activity occurs with serum LD5. Its activity, by comparison, is stable over the wide pH range of 9·00 to 9·55. It should be noted, however, that the protein concentration of the purified LD5 used in this experiment is much lower than that present in serum and this may contribute to the decreased stability.

*Comments on the pH optimum*

The broad pH profile at 30°C (figure 5) indicated that the optimum for LD1 in both serum and the purified form was 10·3. The activity increased steadily with the increasing pH until pH 9·6, above which there was a sharp increase in activity until pH 10·3. Above this value, the activity dropped rapidly to zero either due to the instability of NAD$^+$ at very alkaline pH or the instability of the enzyme itself. The optimum pH for LD5 in both serum and the purified form was between 9·2 and 9·5. At pH values greater than 9·6 the activity decreased significantly, reaching zero by 10·5 and 10·8 for the purified and the enzyme in serum, respectively.

The experiments using the narrow pH range at both 30°C and 37°C contributed to the selection of a suitable assay pH value. In these experiments, LD1 in both the purified and serum forms exhibited a small increase in activity with pH until 9·60 above which the increase was more rapid. The patterns were similar at both assay temperatures. At 30°C, the smallest incremental change was between pH 9·40 and 9·55 and at 37°C the smallest incremental change was between pH 9·20 and 9·45. The optimum pH for LD5 in both serum and the purified form at 30°C was between 9·20 and 9·50. At 37°C, the LD5 in serum was stable within this pH range but the purified form lost activity at pH values greater than 9·40 probably due to both the inactivation of the enzyme at the more alkaline pH values and the greater heat sensitivity of the LD5 isoenzyme at the higher assay temperature.

*From the above results, we concluded that the most suitable pH value for the assay of lactate dehydrogenase is 9·40.* This value falls within the pH range where the activity of LD1 has the smallest incremental change, is included in the optimum pH range for LD5 and is close to the pKa value of 9·6 for the NMG buffer. It also is the same as the optimum pH selected independently by the DGKC [11].

*Buffer concentration*

To determine the optimum buffer concentration, the concentration of NMG buffer at pH 9·4 was varied in the assay and the activity measured (figure 8). Initially, this experiment was carried out at 30°C using samples consisting of purified LD1 and LD5 and serum which contained predominantly either LD1 and LD5.

From this experiment, it can be seen that a buffer concentration of 325 mmol/l is suitable for use in the assay

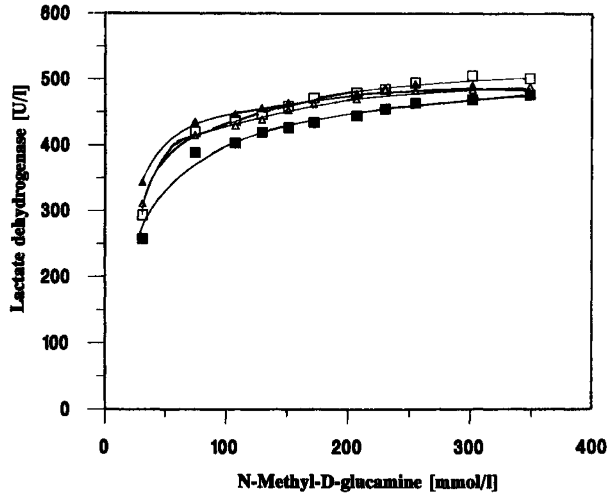

*Figure 8. Effect of buffer concentration on the activity of lactate dehydrogenase at 30°C. The samples that were assayed were purified LD1 (■), purified LD5 (▲), a specimen containing predominantly LD1 (□) and a specimen containing predominantly LD5 (△). The substrate concentrations were 50 mmol/l lactate and 10 mmol/l NAD$^+$. The assay was initiated by the addition of NAD$^+$.*

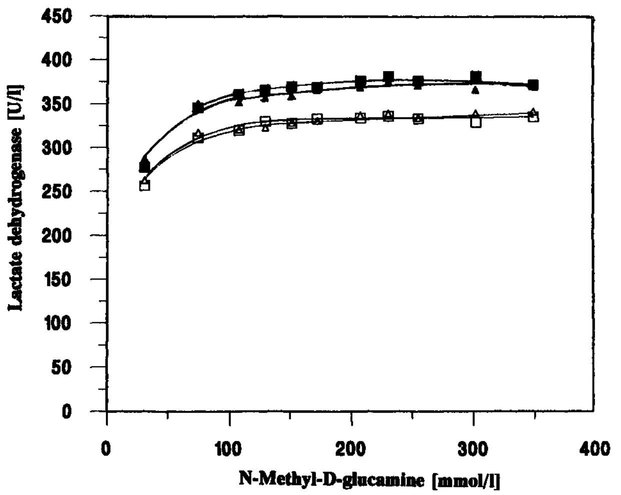

*Figure 9. Effect of buffer concentration on the activity of lactate dehydrogenase at 37°C. The samples that were assayed were purified LD1 (■), purified LD5 (▲), a specimen containing predominantly LD1 (□) and a specimum containing predominantly LD5 (△). The substrate concentrations were 50 mmol/l lactate and 10 mmol/l NAD$^+$. The assay was initiated by the addition of NAD$^+$.*

of lactate dehydrogenase. In addition, the same experiment, although using different serum specimens, was carried out at 30°C (figure 9). This showed that 325 mmol/l is also a suitable buffer concentration at this temperature. Other experimental work carried out to determine the effect of substrates on the pH value of the buffer also indicated that at this concentration, changes in pH are minimal.

### *Effect of temperature of buffer preparation on assay pH*

As was shown previously, varying the temperature has an effect on the pH of the buffer. This means that the final pH value at 30°C or 37°C will depend on the temperature at which the buffer is prepared. Although buffers should be prepared at the temperature at which the assay is to be carried out, in practice they are often prepared at room temperature (24°C). We have found that for the assay to be carried out at pH 9·40 at 30°C and 37°C, the buffer needs to be prepared at room temperature at pH 9·50 and 9·63, respectively.

### *Effect of buffer concentration on pH*

It is important that the change in pH due to the effect of temperature and the addition of reaction components is kept to a minimum. We have tested this by preparing the buffer at various concentrations, incubating at both 30°C and 37°C and monitoring the change due to the addition of the reaction components (figure 10).

The results show that at a concentration of 325 mmol/l NMG, the change in pH is at a minimum.

### *Sample volume fraction*

The sample volume fraction optimum for the lactate dehydrogenase assay was determined by assaying increasing amounts of enzyme. The results (figure 11) show that

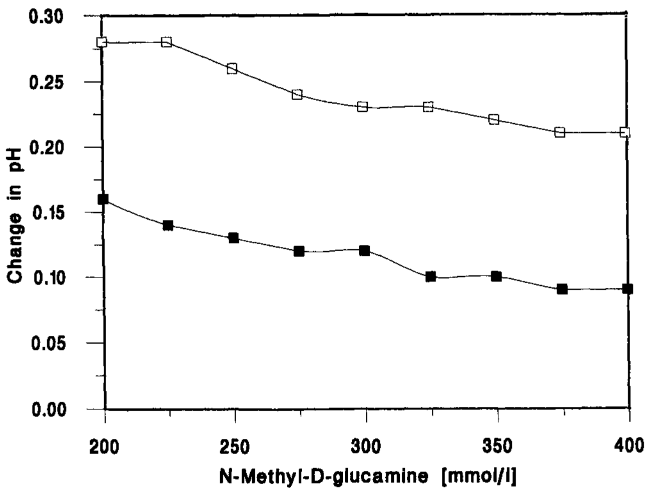

*Figure 10. Effect of buffer concentration on the pH change at 30°C (■) and 37°C.*

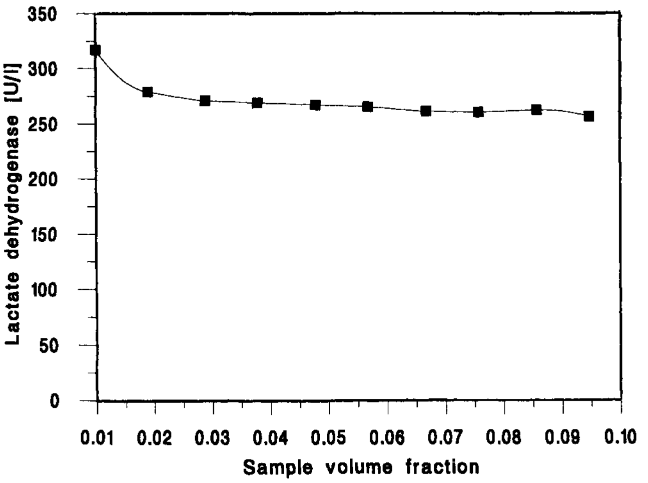

*Figure 11. Effect of increasing the sample volume fraction on the activity of lactate dehydrogenase at 30°C. The sample assayed was a serum containing predominantly LD1 (■).*

there is little effect of the sample volume fraction on the assay between 0·03 and 0·08 and that 0·05 (1:21) is suitable for use in this assay.

### *Sample stability*

To determine the stability of lactate dehydrogenase in serum when incubated at pH 9·4, the enzyme was incubated in NMG buffer for time intervals ranging from 0 to 25 min. At the end of each incubation, the activity was measured by adding the other assay components. Both serum containing predominantly either LD1 and LD5 were rested at 30°C and 37°C (figure 12). Both samples showed no change in activity with time indicating that the enzyme was stable under these conditions.

### *Stability of NAD$^+$*

One of the factors in assaying lactate dehydrogenase is the stability of NAD$^+$. We have studied this by preparing NAD$^+$ in various solutions and then measuring the effect

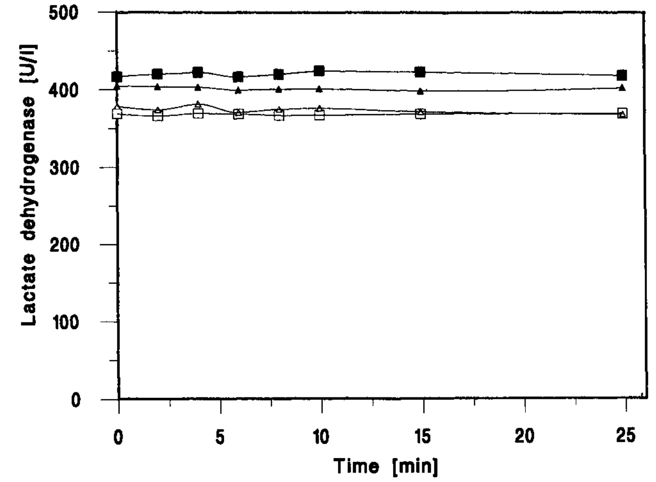

*Figure 12. Stability of lactate dehydrogenase in 325 mmol/l NMG buffer, pH 9·4. Samples were incubated at either 30°C (filled symbols) or 37°C (open symbols) and then removed for assay at selected times. The samples were serum which contained predominantly either LD1 (■, □) or LD5 (▲, △).*

of storage at 4°C on the change in absorbance of the solution and the effect on enzymic activity. Besides choosing various concentrations of NMG, imidazole was also tested because this was recommended by the DGKC as the buffer of choice for preparing $NAD^+$. All the buffers were prepared to pH 9·50 at room temperature (24°C) and then the $NAD^+$ added to give a final concentration of 100 mmol/l $NAD^+$. The resulting $NAD^+$ starting solutions had pH values of 9·00 for 417 mmol/l NMG, 7·95 for 275 mmol/l NMG, 5·38 for 250 mmol/l NMG, 4·24 for 200 mmol/l NMG, 3·53 for 100 mmol/l, 6·03 for 100 mmol/l imidazole and 2·81 for water.

The results (figures 13(*a*) and (*b*) show that the $NAD^+$ reagent remains stable for at least 12 days at 4°C as long as the pH is less than 6·0. Above pH 7·0, $NAD^+$ begins to decompose causing an increase in the absorbance of the solution and a decrease in activity of the lactate dehydrogenase reaction. Our results also indicate that there is no difference in stablity when the $NAD^+$ is prepared in NMG buffer, imidazole buffer or water. For ease of preparation, we recommended using an aqueous solution of $NAD^+$.

*Note.* The pH of an aqueous solution of 100 mmol/l $NAD^+$ is 2·81 only when the $NAD^+$ is in the free acid form. If the lithium salt of $NAD^+$ is used, the resulting pH of the solution may preclude its use in this assay.

*Effect of reaction initiation*

It has been recommended that the reaction be initiated using $NAD^+$ because this minimizes the non-linearity of the reaction curve. We have also tested the effect of initiating the reaction with either sample or lactate and have shown that this does not significantly alter the measured activity when using the recommended conditions.

### Determination of $K_m$ for $NAD^+$ and lactate

In the assay of most enzymes, the concentration of the substrates are chosen so that they are saturating. This is normally done by determining the $K_m$ values of the substrates and then using concentrations $\geqslant 20\ K_m$ (at this concentration of substrate, the catalytic activity should be greater than 95% of the theoretical maximum). The $K_m$ values for the substrates have been determined using zero order kinetics. To determine the $K_m$ values for $NAD^+$ and lactate, the $NAD^+$ concentration was varied at fixed concentrations of lactate.

The experiment was carried out at 30°C on the Cobas

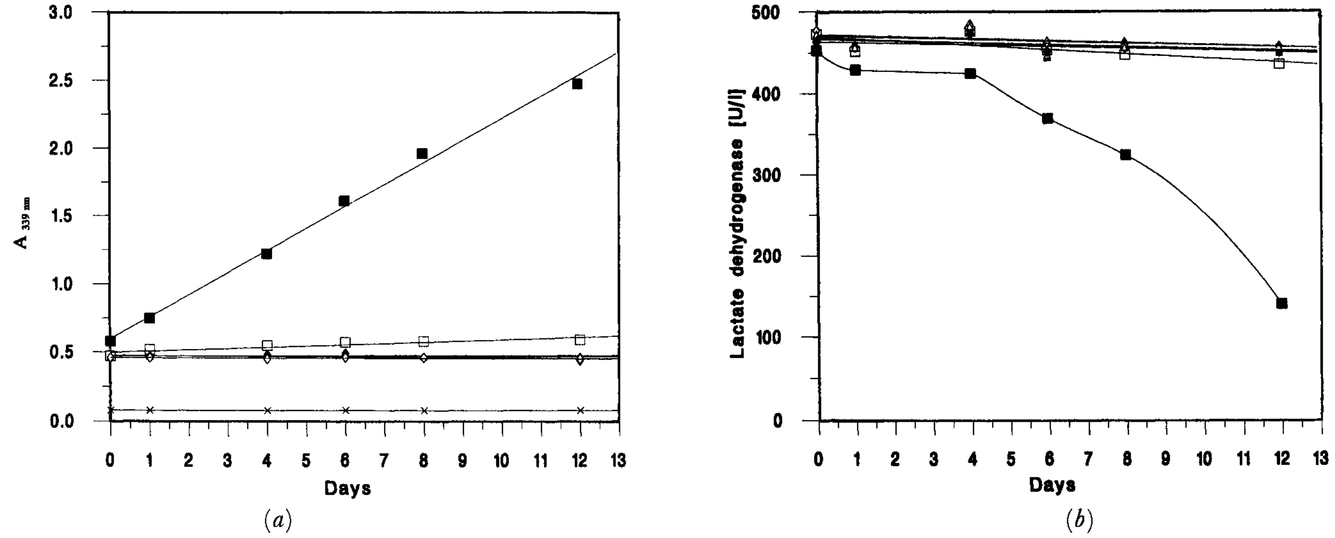

*Figure 13(a) and (b). Effect of various solutions on the stability of $NAD^+$ as measured at 339 nm (a) and the effect of these $NAD^+$ solutions on the activity of lactate dehydrogenase (b). The solutions in which $NAD^+$ was prepared were 417 mmol/l NMG ■, 275 mmol/l NMG □, 250 mmol/l NMG ▲, 200 mmol/l NMG △, 100 mmol/l NMG, ◆, 100 mmol/l imadazole ◇ and water x. The absorbance at 339 nm was measured using a 1 in 10 dilution of each $NAD^+$ solution. Solutions were stored at 4°C.*

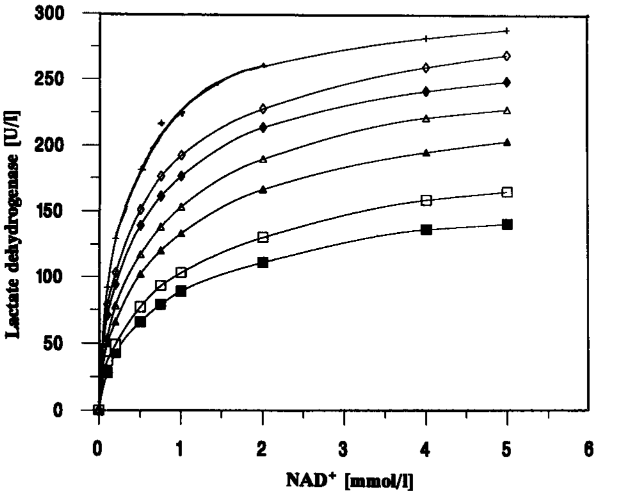

*Figure 14. Activity profiles for LD1 showing the effect of varying NAD$^+$ concentration at fixed concentrations of lactate. The lactate concentrations were 1·5 mmol/l (■), 2·0 mmol/l (□), 3.5 mmol/l (▲), 5 mmol/l (△), 7·5 mmol/l (◆), 10 mmol/l (◇) and 20 mmol/l (+). The buffer concentration used in these experiments was 150 mmol/l NMG, pH 9·4.*

Bio. The buffer/lactate solution was incubated with purified LD1 for 3 min, after which time the NAD$^+$ was added and the reaction monitored between 40 and 200 seconds. This short time period was chosen to ensure that the reaction was monitored over a linear portion of the reaction. The plot of the initial reaction velocity versus the NAD$^+$ concentration at varying levels of lactate are shown in figure 14.

This data can then be transformed into reciprocals and replotted as shown in figure 15.

The replot gives a series of straight lines which intercept at a single point. This is the expected pattern for a sequential mechanism for a bireactant system. Implicit in this mechanism is that all substrates must be present simultaneously at the active site of the enzyme before product formation can occur.

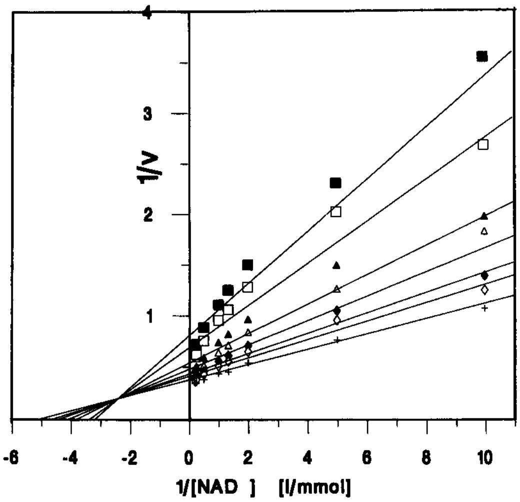

*Figure 15. Double reciprocal plots of the data for LD1 shown in figure 14. The lactate concentrations were 1·5 mmol/l (■), 2·0 mmol/l (□), 3·5 mmol/l (▲), 5 mmol/l (△), 7·5 mmol/l (◆), 10 mmol/l (◇) and 20 mmol/l (+).*

The rate equation describing this mechanism is:

$$v = \frac{V_{max}AB}{K_a'K_b + K_bA + K_aB + AB} \tag{1}$$

When $1/v$ is plotted versus 1/A (where A is the NAD$^+$ concentration and B the lactate concentration) the plot will be a series of straight lines described by the following rate equation:

$$1/v = \frac{1}{V_{max}}\left(\frac{K_a'K_b}{AB} + \frac{K_b}{B} + \frac{K_a}{A} + 1\right) \tag{2}$$

Although the various constants described in equation 2 can be determined by replotting the slopes and intercepts from the double reciprocal plot, in this case the data was fitted to a reiterative computer program which was able to fit the data to equation 2 [14]. From this, the following values were obtained:

$$K_{lactate} = 2{\cdot}0 \pm 0{\cdot}4 \text{ mmol/l}$$

$$K_{NAD^+} = 0{\cdot}18 \pm 0{\cdot}03 \text{ mmol/l}$$

From these kinetic constants, suitable concentrations for optimal activity would be 40 mmol/l for lactate and 3·6 mmol/l for NAD$^+$ (20 × Km), which compares favourably with the selected assay concentrations of 50 mmol/l and 10 mmol/l, respectively.

We have also carried out similar experiments with purified LD5 (figures 16 and 17).

These data were replotted in the double reciprocal form (figure 17) and analysed as described for LD1.

When fitted to equation (2), the following kinetic parameters were obtained:

$$K_{lactate} = 5{\cdot}6 \pm 1{\cdot}2 \text{ mmol/l}$$

$$K_{NAD^+} = 0{\cdot}22 \pm 0{\cdot}06 \text{ mmol/l}$$

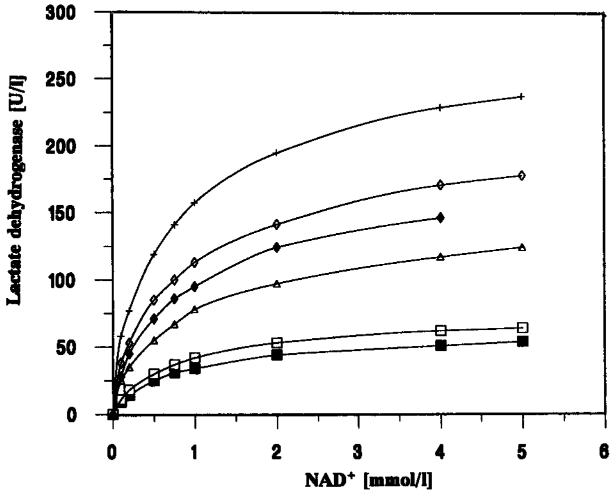

*Figure 16. Activity profiles for LD5 showing the effect of varying NAD$^+$ concentration at fixed concentrations of lactate. The lactate concentrations were 1·5 mmol/l (■), 2·0 mmol/l (□), 3·5 mmol/l (▲), 5 mmol/l (△), 7·5 mmol/l (◆), 10 mmol/l (◇) and 20 mmol/l (+). The buffer concentration used in these experiments was 150 mmol/l NMG, pH 9·4.*

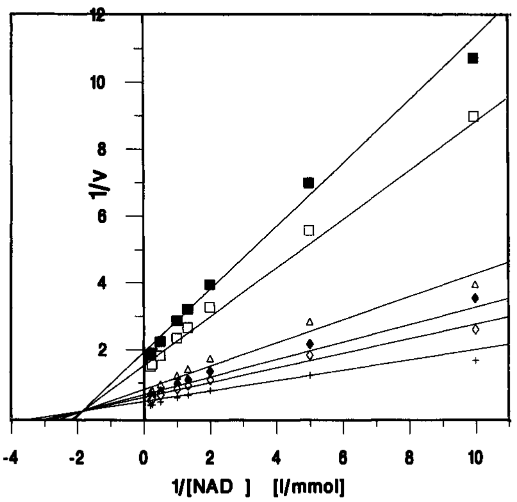

*Figure 17. Double reciporcal plots of the data for LD5 shown in figure 16. The lactate concentrations were 1·5 mmol/l (■), 2·0 mmol/l (□), 3·5 mmol/l (▲), 5 mmol/l (△), 7·5 mmol/l (◆), 10 mmol/l (◇) and 20 mmol/l (+).*

The $K_m$ value for $NAD^+$ obtained for LD5 is slightly greater than that obtained for LD1 (0·22 mmol/l compared to 0·18 mmol/l) whereas the $K_m$ for lactate is considerably greater (5·6 mmol/l compared to 2·0 mmol/l). Thus, to achieve the theoretical maximum catalytic activity for LD5, a lactate concentration of greater than 100 mmol/l would be required.

From previous work and that of others, it is clear that the lactate to pyruvate reaction is curvi-linear. The main reason for non-linearity is thought to be the formation of dead-end complexes during the reaction as depicted in the following mechanism [15]:

E ⇌ E-NADH ⇌ E-NADH-Pyruvate

⇅ Lactate

E-NADH-Lactate

⇌ E-NAD-Lactate ⇌ E-NAD ⇌ E

⇅ Pyruvate

E-NAD-Pyruvate

E-NADH-lactate and E-NAD-pyruvate are dead-end complexes. The formation of these dead-end complexes also suggested that using 20 × $K_m$ as the optimum substrate concentration may compromise the activity, because a greater concentration of substrate would be required to saturate due to the amount bound up in the dead-end complex.

This proposal was tested by fixing the lactate concentration at 50 mmol/l and measuring the activity at varying $NAD^+$ concentrations. This was carried out at both 30°C and 37°C to determine whether the temperature has an effect on the concentration required for maximum activity (figure 18).

The results shows that the formation of the dead-end complexes have an effect on the $NAD^+$ concentration required for saturation. Using the Km value determined for $NAD^+$ in the previous experiments it would be expected that a concentration of 5 mmol/l (>20 Km) would be optimal. However, as can be seen in figure 18, this is not the case and a significantly greater concentration is required.

*A concentration of 10 mmol/l $NAD^+$ was selected as optimal.*

A similar experiment was carried out to determine whether the kinetics of the enzyme reaction has an effect on the concentration of lactate required for optimal activity (figure 19).

The results indicate that a concentration of 50 mmol/l is optimal for LD1 both in the serum and purified form, which agrees with the results obtained from previous kinetic experiments. Above this concentration, there is substrate inhibition. As can be seen in figure 19, this concentration is also optimal for LD5 in serum but for purified LD5, a lactate concentration greater than 100 mmol/l would be required. Even though a lactate concentration of 50 mmol/l is sub-optimal for purified LD5, the activity obtained is still greater than 90% of the maximum.

*A lactate concentration of 50 mmol/l was selected as optimal.*

### Response Surface Optimization

The conditions for the measurement of lactate dehydrogenase have also been investigated using Response Surface Optimization [16]. This technique requires simultaneous variation of the component concentrations with subsequent computer analysis of the responses obtained at the defined reaction conditions. Factorial experimentation at varying pH, buffer concentration, $NAD^+$ and lactate concentrations were conducted at 30°C using sera containing either predominantly LD1 or LD5. Theoretical response surfaces were computed by fitting a second-order polynomial equation using least squares regression analysis techniques. The data were analysed independently by two laboratories (Service de Biochimie, Hopital Debrousse, Lyons, France and the Reference Laboratory, Eastman Kodak Company, Rochester, New York, USA). The results from the response surface optimization experiments are shown in figure 20 (LD1) and 21 (LD5) and the results from the two laboratories are summarized in table 4.

The analysis showed there is a range of concentrations for both lactate and $NAD^+$ which give little variation in LD activity: This range is 26 to 50 mmol/l for lactate and 10 to 16 mmol/l for $NAD^+$. The analysis also showed there is a negative interaction between the optimal concentrations of lactate and $NAD^+$, that is, as the assay lactate concentration is increased, the optimal concentration for $NAD^+$ is lowered, and vice versa. Therefore, at the chosen lactate concentration of 50 mmol/l, the optimal concentration for $NAD^+$ is 10 mmol/l rather than 16 mmol/l. As can be seen from the univariate experiment (figure 18), an $NAD^+$ concentration of 10 mmol/l is close to the optimum. In addition, the reagent blank reading and the

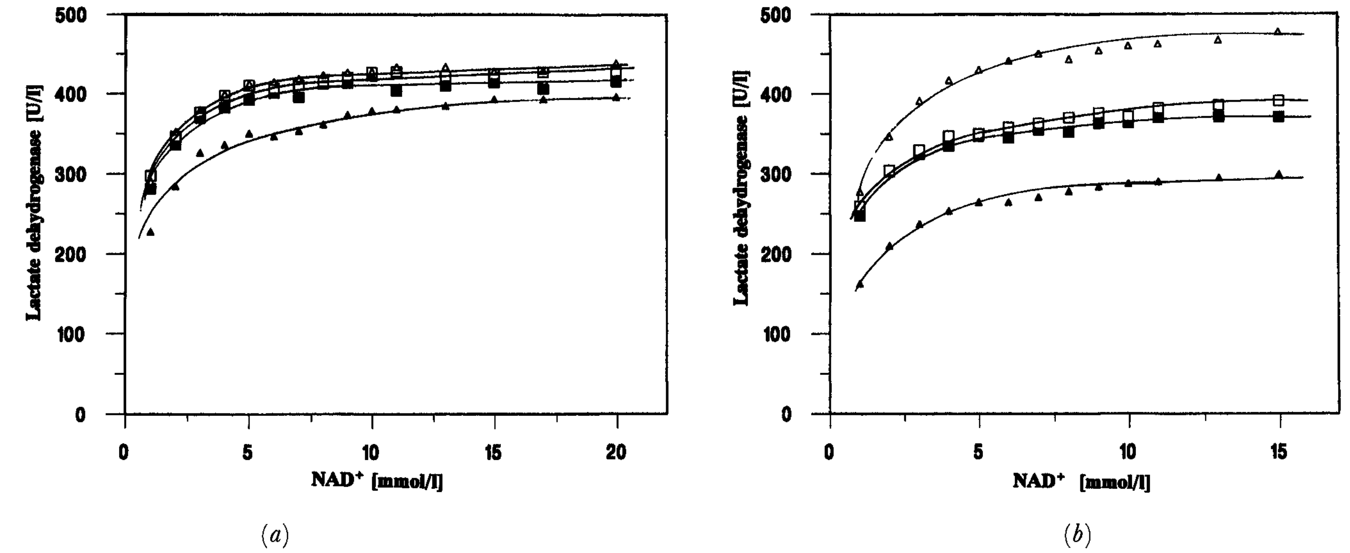

*Figure 18. Effect of varying $NAD^+$ concentration at 50 mmol/l lactate at 30°C (a) and 37°C (b). The samples assayed were purified LD1 (■), purified LD5 (▲), a specimen containing predominantly LD1 (□) and a specimen containing predominantly LD5 (△). The assay was initiated by the addition of $NAD^+$. The concentration of the NMG buffer was 325 mmol/l, pH 9·4. Different enzymes samples were used for the experiments at 30°C and 37°C.*

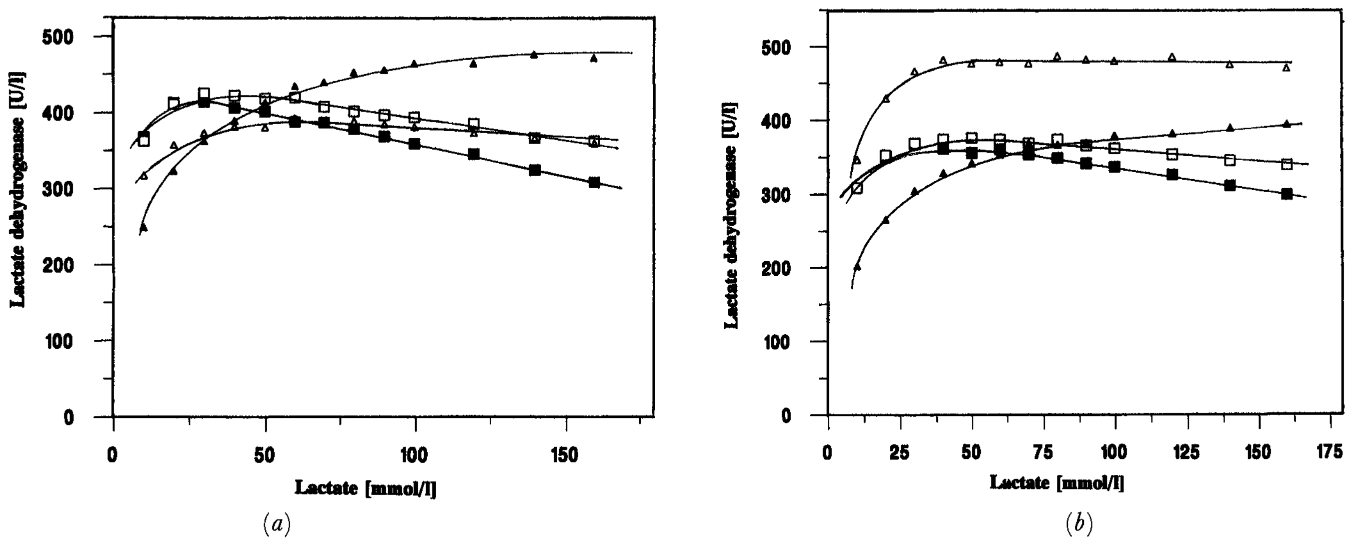

*Figure 19. The effect of varying lactate concentration at 10 mmol/l $NAD^+$ at 30°C (a) and 37°C (b). The reaction was initiated by the addition of $NAD^+$. The samples that were assayed were purified LD1 (■), purified LD5 (▲), a specimen containing predominantly LD1 (□) and a specimen containing predominantly LD5 (△). The concentration of the NMG buffer was 325 mmol/l, pH 9·4. Different enzyme samples were used for the experiments at 30°C and 37°C.*

*Table 4. Optimal values for assay conditions as determined by surface response optimization.*

| | LD1 | | LD5 | | |
|---|---|---|---|---|---|
| | Rochester | Lyons | Rochester | Lyons | Recommended |
| Buffer | 303·5 | 310 | 273 | 340 | 325 |
| pH | 9·90 | 9·90 | 9·74 | 9·8 | 9·4 |
| $NAD^+$ | 15·2 | 16 | 11·7 | 13·6 | 10 |
| Lactate | 37·0 | 35 | 53·5 | 50 | 50 |

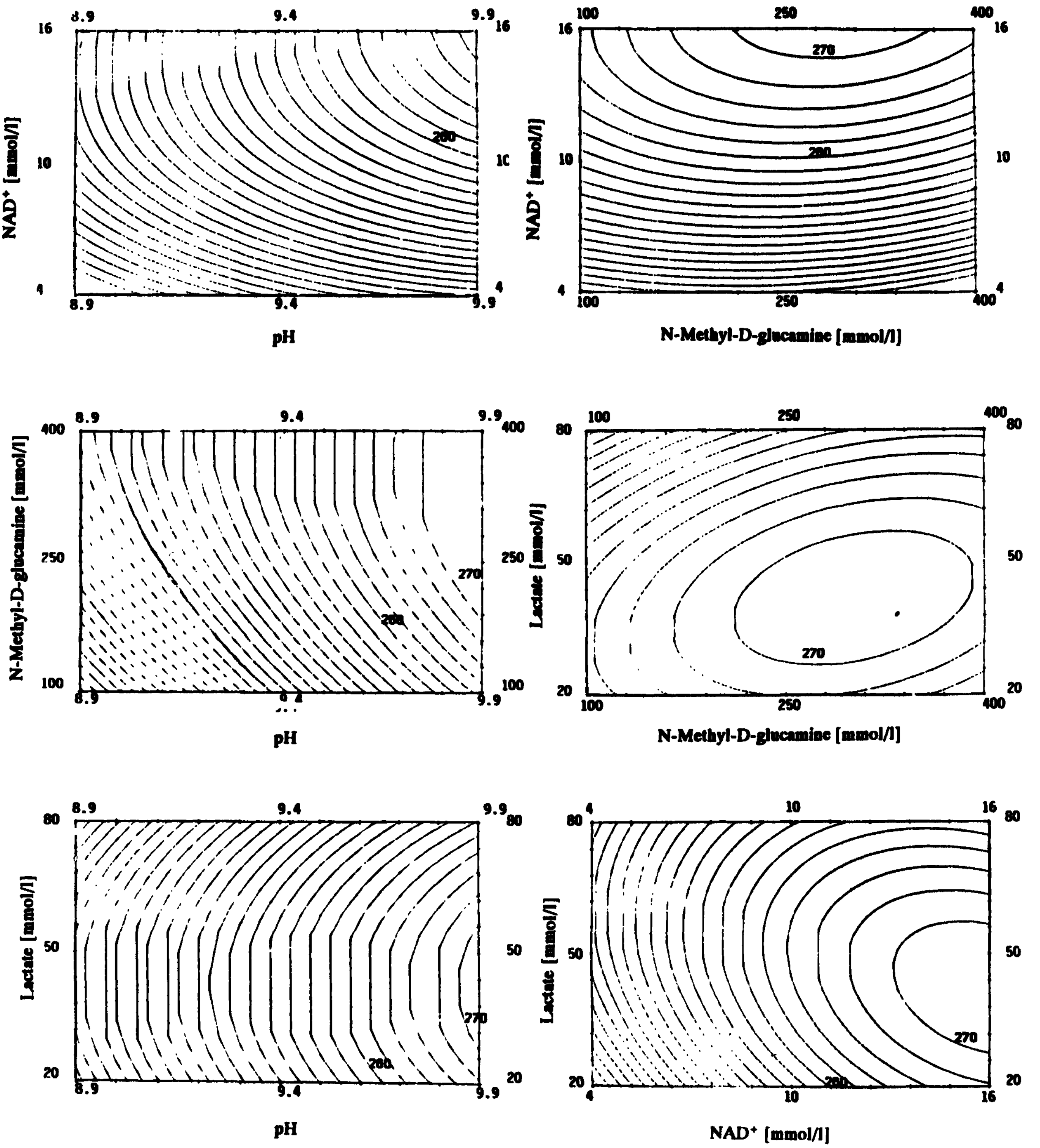

*Figure 20. Optimiazation of using RSO of pH, lactate and NAD$^+$ concentration for human serum containing predominantly LD1. Values used for these experiments were pH (8·9, 9·4, 9·9), lactate concentration (20, 50, 80), NAD$^+$ (4, 10, 16) and buffer concentration (100, 250, 400). Isoactivity contour lines are drawn at intervals of 2 U/l.*

initial absorbance of the assay mixture are more acceptable at the lower NAD$^+$ concentration of 10 mmol/l.

The RSO analysis defined that pH values between pH 9·7 and 9·9 were optimal for the assay. Unfortunately, NAD$^+$ is unstable under these conditions, and therefore, as explained previously, a value of 9·4 was selected as the most practical value to use. Even with these compromises, the assay still gives greater than 95% of the theoretical optimal activity for both LD1 and LD5.

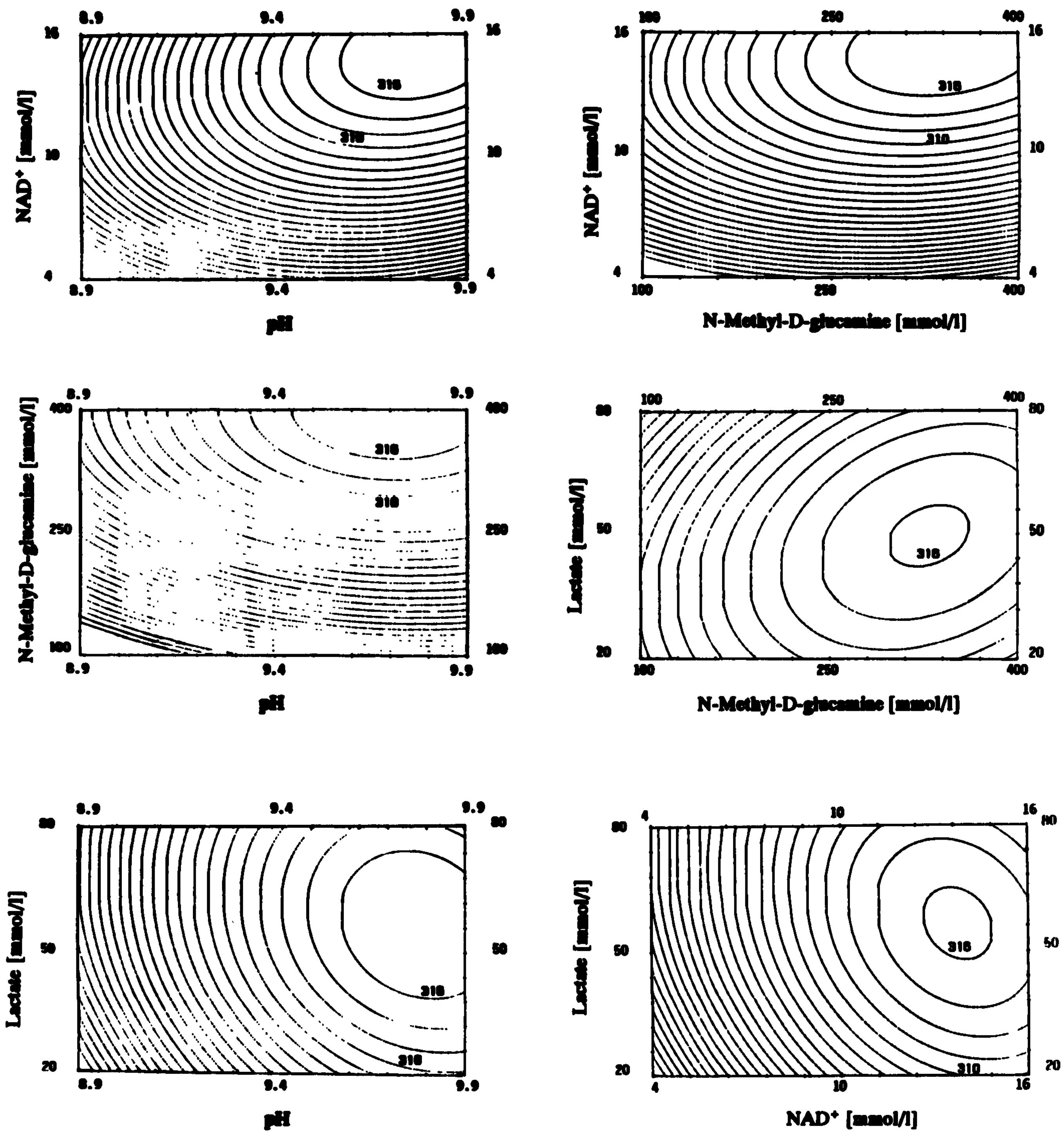

*Figure 21. Optimization using RSO of pH, lactate and NAD$^+$ concentrations for human serum containing predominantly LD5. Values used for these experiments were pH (8·9, 9·4, 9·9), lactate concentration (20, 50, 80), NAD$^+$ (4, 10, 16) and buffer concentration (100, 250, 400). Isoactivity contour lines are drawn at intervals of 2 U/l.*

### Dynamic range

The assay dynamic range was determined by assaying a specimen with high activity which had been diluted with saline. The results (figure 22) show that the assay is linear up to 600 U/l. Above this activity the specimen should be diluted.

### Reference interval

Reference ranges at 30°C have not yet been determined for this method. However, a reference range has been determined at 37°C on healthy people selected from recruiting/engagement examinations at the Medical University of Lübeck, Germany. The results

*Table 5. Reference ranges (U/l) for lactate dehydrogenase assayed at 37°C.*

| Group | *N* | Range | Mean | 0·95–reference interval |
|---|---|---|---|---|
| All | 136 | 132–228 | 172 | 135–225 |
| Females | 74 | 133–226 | 172 | 135–214 |
| Males | 62 | 132–228 | 172 | 135–225 |

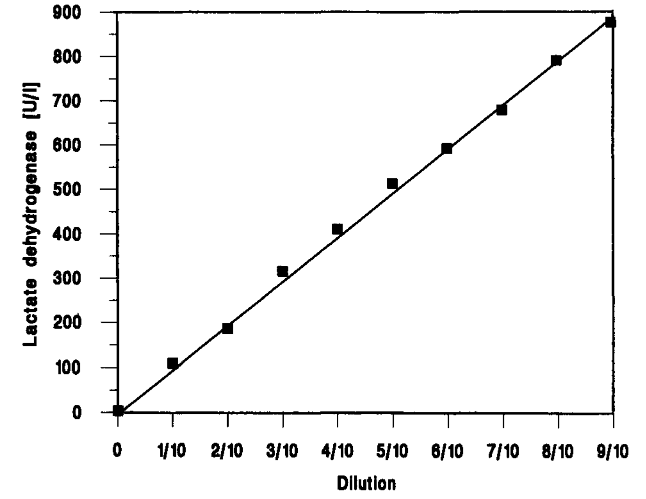

*Figure 22. Dynamic range of lactate dehydrogenase. A serum sample with high activity was diluted with saline and the activity measured.*

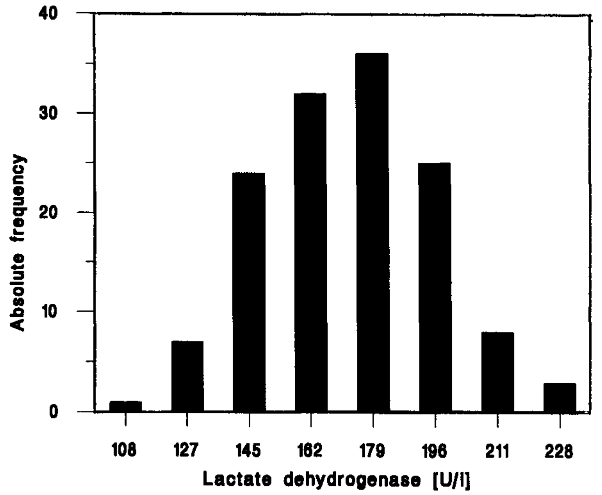

*Figure 23. Distribution of lactate dehydrogenase activity for 136 healthy people (74 females, 62 males assayed at 37°C).*

are shown in Figure 23 and are summarized in table 5.

### Conversion factor 30°C to 37°C

The factor for conversion of results between 30°C and 37°C was determined by assaying 48 specimens at each temperature (figure 24). The specimens were selected for those arriving in the laboratory and not tested for their isoenzyme content. From the results it can be seen that

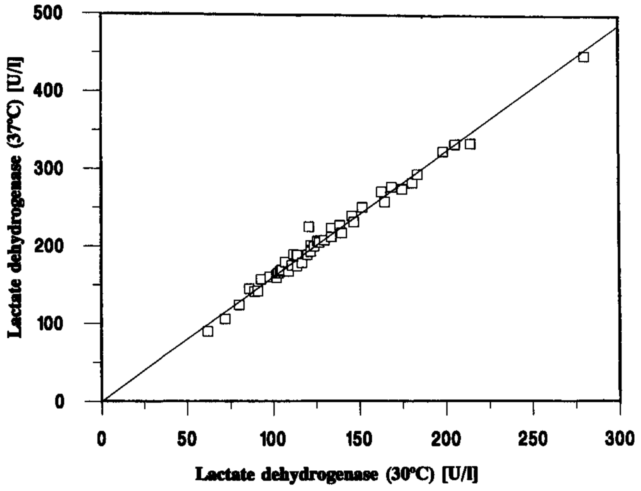

*Figure 24. Comparison of LD activity between 30°C and 37°C. The assays were carried out on the Cobas Bio using the conditions and concentrations as recommended.*

there is an excellent correlation between the recommended method assayed at the two temperatures indicating that it is suitable for use at both 30°C and 37°C. The conversion is

$$(\text{activity})_{37} = 1{\cdot}596\ (\text{activity})_{30}.$$

Although it is not recommended that factors be used routinely to convert activities between different temperatures, it does give an indication of their relationship.

### Acknowledgements

We wish to thank Dr Daniel A. Nealon, Director, Reference Laboratory, Clinical Diagnostics Division, Eastman Kodak Company, Rochester, NY, USA and Drs C. Lahet and I. Maire, Service de Biochimie, Hôpital Debrousse, Lyons, France for analysing the surface response data; and Professor Klaus Lorentz, Institute for Clinical Chemistry, Medical University of Lübeck, Germany for providing the reference intervals. This work was supported by grants from Bayer Diagnostics, Kodak (Aust) Pty Ltd and Boehringer Mannheim (Aust) Pty Ltd and financial assistance from Roche Products Pty Ltd, Beckman Instruments (Aust) Pty Ltd and Abbott Diagnostics Division.

### References

1. Vanderlinde, R., *Annals of Clinical Laboratory Science*, **15** (1985), 13.
2. Buhl, S. N. and Jackson, K. Y., *Clinical Chemistry*, **24** (1978), 828.

3. McComb, R. B., In *Clinical and Analytical Concepts in Enzymology*, edited H. A. Homburger (College of American Pathologists, Skokie, 1983).
4. Clark, P. I., Kostuk, W. J., and Henderson, A. R., *Clinical Chimica Acta*, **36** (1976), 361.
5. Buhl, S. K., Jackson, K. Y., Lubinski, R. and Vanderlinde, R. E., *Clinical Chemistry*, **22** (1976), 22.
6. Bais, R. and Edwards, J. B., *Clinical Chemistry*, **26** (1980), 525.
7. Cabello, R., Lubin, J., Ryrolin, A. M. and Frankel, R., *American Journal of Clinical Pathology* **73** (1980), 253.
8. Bhagaran, N. V., Dorin, J. R. and Scottalivic, A. G., *Arch Pathol Lab med*, **106** (1982), 521.
9. Podlasek, S. J., McPherson, R. A. and Threatte, G. A., *Clinical Chemistry*, **30** (1984), 266.
10. Kato, S., Ishii, H., Horii, K. and Tsuchiya, M., *Clinical Chemistry*, **30** (1984), 1585.
11. Schmidt, E., Henkel, H., Klauke, R., Lorentz, K., Sonntag, O., Stein, W., Weidemann, G. and Gerhardt, W., Working Group on Enzymes of the Germany Society of Clinical Chemistry, *European Journal of Clinical Chemistry and Clinical Biochemistry*, **30** (1992), 787.
12. Novoa, W. B., Winer, A. D., Glaid, A. J. and Schwert, G. W., *Journal of Biological Chemistry*, **234** (1959), 1143.
13. Schmidt, E., Henkel, H., Klauke, R., Lorentz, K., Sonntag, O., Stein, W., Weidemann, G. and Gerhardt, W., Working Group on Enzymes of the German Society of Clinical Chemistry, *European Journal of Clinical Chemistry and Clinical Biochemistry*, **30** (1992) 247.
14. Duggleby, R. G., *Annals of Biochemistry*, **110** (1981), 9.
15. Howell, B. F., McCune, S. and Schaffer, R., *Clinical Chemistry*, **25** (1979) 269.
16. London, J. W. In *Clinical and Analytical Concepts in Enzymology*, edited by H. A. Homburger (College of American Pathologists, Skokie, 1983).

